# Advancing discussion of ethics in mixed methods health services research

**DOI:** 10.1186/s12913-021-06583-1

**Published:** 2021-06-15

**Authors:** Nicole A. Stadnick, Cheryl N. Poth, Timothy C. Guetterman, Joseph J. Gallo

**Affiliations:** 1 Department of Psychiatry, University of California San Diego, La Jolla, CA USA; 2 University of California San Diego Altman Clinical and Translational Research Institute Dissemination and Implementation Science Center, La Jolla, CA USA; 3 Child and Adolescent Services Research Center, San Diego, CA USA; 4 Department of Educational Psychology, University of Alberta, Edmonton, AB Canada; 5 Department of Family Medicine, University of Michigan, Ann Arbor, MI USA; 6 Mixed Methods Research Training Program, Department of Mental Health, Johns Hopkins University Bloomberg School of Public Health, Baltimore, MD USA

**Keywords:** Mixed methods, Research ethics, Health services research

## Abstract

**Background:**

To describe the ethical issues and experiences of scientists conducting mixed methods health services research and to advance empirical and conceptual discussion on ethical integrity in mixed methods health research.

**Methods:**

The study was conducted with 64 scholars, faculty and consultants from the NIH-funded Mixed Methods Research Training Program (MMRTP) for the Health Sciences. This was a cross-sectional study. Survey results were analyzed using descriptive statistics to characterize responses and open coding to summarize strategies about eight ethical mixed methods research issues. Respondents completed an online survey to elicit experiences related to eight ethical issues (informed consent, confidentiality, data management, burden, safety, equitable recruitment, communication, and dissemination) and strategies for addressing them.

**Results:**

Only about one-third of respondents thought their research ethics training helped them plan, conduct, or report mixed methods research. The most frequently occurring ethical issues were participant burden, dissemination and equitable recruitment (> 70% endorsement). Despite occurring frequently, < 50% of respondents rated each ethical issue as challenging. The most challenging ethical issues were related to managing participant burden, communication, and dissemination. Strategies reported to address ethical issues were largely not specific or unique to mixed methods with the exception of strategies to mitigate participant burden and, to a lesser degree, to facilitate equitable recruitment and promote dissemination of project results.

**Conclusions:**

Mixed methods health researchers reported encountering ethical issues often yet varying levels of difficulty and effectiveness in the strategies used to mitigate ethical issues. This study highlights some of the unique challenges faced by mixed methods researchers to plan for and appropriately respond to arising ethical issues such as managing participant burden and confidentiality across data sources and utilizing effective communication and dissemination strategies particularly when working with a multidisciplinary research team. As one of the first empirical studies to examine mixed methods research ethics, our findings highlight the need for greater attention to ethics in health services mixed methods research and training.

**Supplementary Information:**

The online version contains supplementary material available at 10.1186/s12913-021-06583-1.

## Background

Mixed methods research (MMR) is a methodological approach that involves systematic collection, analysis, and integration of both quantitative and qualitative data for the purpose of developing a more comprehensive understanding of a research question than might be garnered through quantitative or qualitative methods alone [1]. Mixed methods health services research has grown significantly over the past few decades as evidenced by a growing trend in use of mixed methods in National Institutes of Health proposals [2, 3] and in health services journals [4]. In response to the recognition of the value of mixed methods in health services research, resources for guiding MMR development, conduct and dissemination have proliferated, including textbooks, courses, formal training programs [5] and the NIH Office of Behavioral and Social Sciences Research Best Practices for Mixed Methods Research in the Health Sciences [1, 6].

Within excellent guiding MMR resources is a notable absence of detailed discussions about ethics and the practice of ethical MMR (i.e., ethical integrity). That little conceptual and virtually no empirical work has been conducted to advance guidance in mixed methods ethics may be surprising given the need for researchers to gain study approvals from various ethical review boards (i.e., institutional and community). The current state of the field is aptly described by Preissle and colleagues [7]: “ethics in MMR remains uncharted.” In writing this paper we respond to their call to “encourage others to forge their own paths, explore the terrain from their own perspectives, and contribute to our ongoing and shared enterprise.” We were surprised that our comprehensive literature review did not identify any empirical studies on MMR ethics to advance the practice of ethical MMR. The NIH Best Practices for MMR [1, 7] explains that use of MMR may introduce additional ethical issues that arise conducting solely quantitative or qualitative research. Examples include MMR requiring collection of identifying information from participants, contacting participants at later points for additional information, and placing a higher burden (time, resources) on participants than a single-method approach. Thus, the well-being of participants must be balanced with potential risks involved with asking more of participants including their time and identification. Attention to ethical issues in MMR is essential because MMR is complex, arguably more so than single-method research, and complexity increases potential ethical pitfalls. An example of complexity is that MMR often includes multidisciplinary teams from diverse disciplines (e.g., medical anthropology, public health, health services, psychology), all of which may have a unique code of ethics and disciplinary cultures that affect conduct of research.

While the NIH Best Practices for MMR in the health sciences briefly mentioned three ethical considerations (i.e., collecting identifying information from participants; the need to contact participants at a later time for additional interviews; higher participant burden in MMR), practical guidance was lacking. Most MMR textbooks identify the need to attend to ethical considerations [1, 7–13] but only Preissle et al. describe ethical issues from a mixed methods perspective that encompasses design, sample selection, data collection, relationships of researchers with participants and stakeholders, and presentation of research findings. Curry and Nunez-Smith focus on advice for submitting mixed methods health services applications to institutional review boards [9]. While the literature on ethics in quantitative or qualitative single-method studies is robust (e.g., [14–16]), focused attention on MMR ethics and the practice of ethics in MMR is a critical need for the field.

The need for greater focus on practical guidance on ethics in MMR is borne out in our data from the NIH-funded Mixed Methods Research Training Program (MMRTP) for the Health Sciences. The MMRTP is a 1-year fellowship that provides intensive training to competitively selected scholars in MMR through development of a NIH grant proposal [5, 17, 18]. First, the need for additional training beyond the fellowship on ‘how’ ethical strategies are applied to MMR was noted by MMRTP scholars via a structured pre/post survey evaluation. Scholars reported a need to improve skills related to the ethical principles of consent and recruitment before program participation. However, scholars reported no change in their self-reported ability to “define or explain” or “apply to practical problems” the ethical principles of consent nor of recruitment following MMRTP participation [17]. Scholars’ self-ratings offer initial support for enhanced attention to MMR ethical training in the MMRTP. Second, we analyzed NIH summary statements from grants submitted by MMRTP scholars and faculty and identified three inductive themes that emerged from the study section reviewer comments regarding ethical concerns. These included: (1) subject burden of participating in both qualitative and quantitative data collection; (2) participant confidentiality and anonymity, particularly risk of participant identification during the qualitative phase of the project or if video recordings were a data source; and (3) informed consent procedures that may require more explanation and thus be time-intensive [19]. Thus, our previous work with the MMRTP motivated our current study focus on understanding ethical considerations derived from practical experience in carrying out MMR.

We leveraged the network of scholars, faculty and consultants in the NIH-funded MMRTP for the Health Sciences to ask about ethical issues in MMR and what strategies investigators use to mitigate them. To address the empirical gaps in MMR and practice, this study had two primary objectives: (1) describe the ethical issues and experiences scientists conducting mixed methods health research; and (2) advance discussion about practices that promote MMR integrity, including the need for mixed-methods tailored research ethics training programs. Our overarching goal was to offer practical considerations to promote ethical research integrity to those pursuing MMR based on the experiences of a sample of faculty and scholars with a special interest in MMR, and to stimulate further research on ethics in MMR.

## Methods

### Mixed methods research training program

The context for our study sample was the MMRTP, an NIH-funded training grant designed to provide intensive training in mixed methods research to scholars through the development of an NIH grant proposal. Competitively selected scholars (primarily early-career faculty) participate in 1) a three-day in-person retreat with lectures and interactive discussions about their projects, 2) webinars on mixed methods topics, and 3) ongoing support from a mentor selected from network of consultants created for the MMRTP who each have content and mixed methods expertise [5, 17, 18]. Sampling from MMRTP scholars and faculty taps a group who are actively planning and carrying out mixed methods research projects, and so should have diverse experiences across a range of disciplines in the health sciences from which to draw when reflecting on ethical considerations.

### Recruitment

An invitation email was sent on April 13th, 2020 to 104 MMRTP scholars (2015–2019 cohorts), faculty and consultants. Three reminder e-mails plus two personalized e-mails from the MMRTP Director were distributed between the initial invitation and June 8th, 2020. Each email included a brief overview of the project and our outreach, an invitation to complete the survey, and an offer for individuals to share their contact information to receive the final report. The final sample included 64 participants, a response rate of 62%. The Institutional Review Board of the Johns Hopkins University deemed the study exempt. All methods were carried out in accordance with relevant guidelines and regulations. No experimental protocols were used to conduct this study.

### Assessment strategy

The online survey was designed to be completed in approximately 15 min. Our assessment instrument had three sections: (1) questions about the respondent’s experience with MMR and research ethics; (2) inquiry about eight ethical issues the respondent may have encountered in MMR and strategies for mitigating them; and (3) additional demographic information (e.g., focus of research training, professional title, and discipline) in a closing section that invited participants to provide their email to obtain more information (the questionnaire is provided in the Additional file 1). We recognize that ethical principles such as respect for autonomy, nonmaleficence, beneficence, and justice underlie criteria for what makes research ethical, and that informed consent alone does not make research ethical [20]. As a practical matter, similar to the approach taken in the chapter by Preissle and colleagues [7] the eight ethical issues were selected based on our collective experience submitting health services and MMR protocols to our local IRBs, the senior author’s experience serving as a member of an IRB, and information typically required to complete IRB applications. These issues were organized and presented in the survey in chronological order across the stages of planning, conducting and reporting of research.

The first section asked about background in MMR, professional experiences (e.g., “I have published a paper using mixed methods”), and training in ethics and whether that training helped in thinking about planning, conducting, and reporting MMR. The second section asked about respondent experiences planning and conducting MMR specific to eight ethical issues: informed consent; equitable recruitment; confidentiality; participant burden; ensuring participant safety; data management; communication between and among the research team (including academic-community partnerships); and dissemination of MMR results. Across issues, respondents were asked to rate (on a four-point scale) how often ethical issues came up (response options ranged from “never” to “always”), how challenging it was to address ethical issue (response options ranged from “not at all challenging” to “very challenging”), what strategies respondents used to mitigate concerns (free text responses), and how effective reported strategies were (response options ranged from “not at all effective” to “very effective”). Qualtrics XM software [21] was used to program and electronically distribute the survey.

### Analysis

Our analysis strategy consisted of two phases. First, we carried out descriptive statistics (frequency distributions) of survey items that focused on experiences conducting MMR and ethical issues encountered. In the second phase, we analyzed all free text responses with open coding. Text responses tended to be short (typically 1 to 3 sentences) and coding was undertaken independently by the authors followed by group discussions. The text responses were analyzed within each ethical issue (informed consent, etc.) and categorized into themes. Similarities and differences across ethical issues were then discussed. Given the focus on MMR research, we paid special attention to responses that were considered by the authors to be referring to MMR. We used a joint display to juxtapose survey results on frequency, challenge, and effectiveness of mitigation strategies for each issue, with open-ended responses on mitigation strategies.

## Results

### Sample background, MMR experience and ethical training

A total of 64 MMRTP scholars, faculty and consultants submitted full survey responses. Although diverse in current professional health-focused disciplines and primary focus of research training, the majority held faculty appointments and had completed mandatory ethics training (Table 1). The most common MMR experiences involved having presented at an MMR meeting (89%), writing an MMR application (84%), publishing an MMR paper (73%) and providing MMR mentoring (73%). Although nearly all respondents reported completing ethics training, only about one-third of respondents reported that their mandatory ethics training was helpful for planning, conducting, and reporting MMR.

**Table 1:** Participant background and ethical training characteristics. Data from the NIH Mixed Methods Research Training Program for the Health Sciences, 2020

Background and Training Characteristics	Frequency (***n*** = 64)	Percentage
**Reported Professional Title**		
Assistant Professor-level faculty	21	33%
Associate Professor-level faculty	14	22%
Full Professor-level	18	29%
Research Scientist	6	10%
Other (i.e., Retired, Director, Clinical Investigator)	4	6%
**Reported Primary Discipline**		
Public Health (including Global and Population Health)	18	29%
Psychology/Psychiatry	9	14%
Medicine (Internal, Family Medicine, General)	8	13%
Anthropology	7	11%
Social Work	5	8%
Nursing	4	6%
Other Health Sciences Fields (i.e., Audiology, Oncology, Healthcare Management; Social and Behavioral Sciences; Nutrition, Pediatrics)	12	19%
**Identified Primary Focus of Research Training**		
Quantitative	41	64%
Qualitative	20	32%
Mixed Methods Research	19	31%
**Research Ethics Training**		
Completed Ethical Conduct of Research Training (ever)	63	98%
Ethics Training was Mandatory	62	97%
**Perceptions of Ethics Training**		
Agreed helped plan MMR	23	36%
Agreed helped conduct MMR	22	34%
Agreed helped report MMR	20	31%
**Experiences with MMR Research (ever)**		
Presented at an MMR meeting	57	89%
Wrote an MMR application that was funded	53	84%
Published an MMR paper	47	73%
Provided MMR mentoring	47	73%
Wrote an MMR thesis or dissertation	13	21%

### Perceptions of frequency, challenge and effectiveness of mitigation strategies

The ratings of frequency, how challenging each ethical issue was to address and perceived effectiveness of reported strategies to address ethical issues are shown in the joint display in Table 2. Of the eight ethical issues examined in the survey, it is noteworthy that six were rated as occurring “Often” or “Always” for more than 60% of all respondents. In particular, participant burden (78.2%) and dissemination of project results (71.9%) were the most frequently encountered whereas informed consent (45.3%) and safety (42.2%) were encountered with the least frequency. Despite the high frequency, respondents largely rated each ethical issue as “not at all” or “somewhat” challenging. The most frequently encountered ethical issues were also rated as “challenging” or “very challenging”; namely, dissemination (46.7%), burden (43.6%), and communication (41.9%). We also examined respondents’ ratings of the effectiveness of their described strategies to address each ethical issue. Of those who reported strategies, the majority (> 50%) rated strategies as “Effective” or “Very Effective” with the exception of communication (50%) and dissemination (41.3%). Strategies rated as most effective were in informed consent (83%), confidentiality (73.8%), data management (65.9%) and burden (62.1%).

**Table 2:**
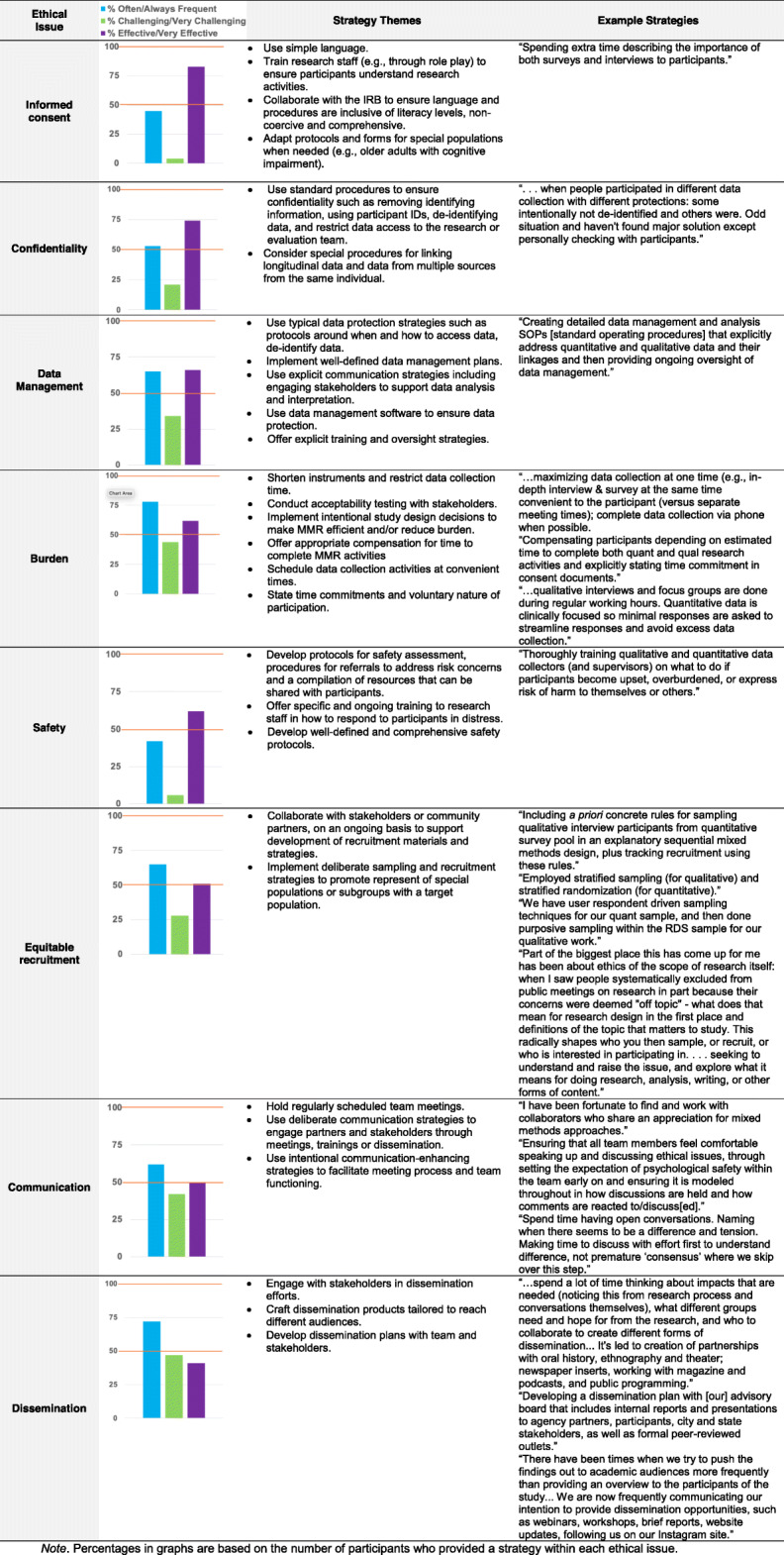
Joint display of strategies to mitigate ethical issues under 8 domains, with percent of respondents who rated the domain frequency, challenge, and effectiveness of strategies to address the domain. Data from the NIH Mixed Methods Research Training Program for the Health Sciences, 2020.

*Note*. Percentages in graphs are based on the number of participants who provided a strategy within each ethical issue

### Specific mitigation strategies

In the following section, we describe the strategies reported by respondents for addressing each ethical issue. We start with describing strategies reported to be standard or typical across research methods and finish with the strategies that were described as unique to MMR. Specifically, strategies to address ethical issues related to informed consent, confidentiality, data management, and safety were described as typical across research methods. Strategies to address participant burden, equitable recruitment, communication, and dissemination were described as having unique relevance to MMR. Strategies (with examples emphasizing mixed methods) are shown with survey ratings in the joint display (Table 2).

#### Informed consent

Respondents reported that they largely used typical strategies to ensure ethical adherence to informed consent procedures such as using simple language and training research staff to ensure that participants thoroughly understand research activities and expectations.

#### Confidentiality

Similar to informed consent, respondents generally reported typical strategies to ensure ethical adherence to participant confidentiality such as removing identifying information, using participant IDs and restricting data access to the research/evaluation team.

#### Data management

Respondents largely described standard data protection strategies such as protocols around when and how to access data, clear and well-defined data management plans, and using specialized data management software to ensure data protection. Some respondents acknowledged the challenge of integrating qualitative and quantitative data in an efficient and optimal manner.

#### Safety

Similar to informed consent and confidentiality, participants described use of typical strategies to ensure ethical adherence to participant safety and wellbeing. These included developing well-defined and comprehensive protocols for safety assessment; procedures for referrals to address risk concerns; developing and offering a compilation of resources that can be shared with participants; and specific and ongoing training of research staff in these safety procedures.

#### Burden

Several unique MMR-specific strategies were described to mitigate participant burden. These included developing brief instruments or revising into shortened instruments; conducting user/acceptability testing (e.g., cognitive interviewing) with stakeholders prior to administration and/or after data collection; making intentional study design modification or choices to make MMR efficient and/or reduce burden; offering appropriate compensation to complete MMR activities; scheduling data collection activities at convenient times and clearly stating time commitments and the voluntary nature of participation in consent documents.

#### Equitable recruitment

Respondents described two primary strategies that acknowledged the complexity of MMR recruitment: (1) collaborating with stakeholders or community partners to support development of recruitment materials and (2) deliberate sampling and recruitment strategies to promote representation of special populations or subgroups within a target population. Examples included using US census data to match sample proportions of sample subgroups, working with methodological experts to develop and ensure recruitment goals, employing a staged/phased recruitment approach to maximize representation, adding multiple sites, and accessing national networks with a more diverse sample.

#### Communication

Respondents emphasized community-engaged and team effectiveness strategies as ways to promote effective communication between and among research and community team members.

#### Dissemination

Strategies to promote ethical dissemination of MMR findings included: engaging with stakeholders in dissemination efforts (e.g., co-author publications/presentations, member checking, share copies of publications with participants); crafting dissemination products tailored to reach different audiences (e.g., through research or policy briefs) through various strategies (e.g., focusing on highly relevant points, using visual displays, ensuring translations in multiple languages); developing dissemination plans with the research team; and relevant stakeholders that included guidance on authorship of products and procedures for reviewing dissemination materials.

## Discussion

Our overarching goal was to describe the experiences of scientists conducting mixed methods health services research to offer practical considerations to encourage discussion on MMR ethics and practices that promote MMR integrity in the field of health services. This sample of MMR scientists reported frequent encounters with ethical issues in MMR, particularly related to participant burden, dissemination, and equitable recruitment. Yet respondents reported significant variability in ratings of the difficulty of addressing ethical issues. For example, fewer than 25% of respondents reported that informed consent, participant confidentiality, and ensuring participant safety were challenging ethical issues to address. Similar levels of variability were shared in ratings of how effective strategies were in addressing ethical issues. While 83% of respondents reported that strategies to address informed consent were effective, only 41% thought strategies to address disseminating project results were effective. Ratings were enhanced by eliciting specific strategies to address ethical issues.

Similar to our approach, in a chapter highlighting ethical issues in their own mixed methods research, Preissle and colleagues [7] considered ethical dimensions with respect to decisions that a mixed methods investigator must make in designing and carrying out a study, from design to presenting the findings. Curry and Nunez-Smith also focused on elements relevant to health services research typically required in IRB applications [9]. Preissle et al. emphasized some aspects of research that we did not, such as the need for reflexivity in use of qualitative methods and relationships with team members from disciplines that may interpret data differently, but for the most part identifying similar ethical issues. In contrast to our work, both sources [7, 9] were based on the deep experiences of the authors rather than a sample of investigators across disciplines and skill levels with a specific interest in mixed methods.

Before discussing our findings in detail, limitations should be mentioned. First, respondents were asked about specific steps in planning or carrying out MMR, and ethical issues encountered throughout the research process. We could not determine whether their strategies actually supported ethical practices. Second, our methodology was limited to self-report, which has inherent drawbacks including subjective response bias in the forms of social desirability or varying comfort levels in sharing experiences. We did not examine proposals, IRB applications, or observe practices. Third, our sample was based on a limited number of investigators who participated in a national, competitive MMR training program. It is noteworthy that our respondents were highly engaged in mixed methods activities in the health sciences in that they had sought out training in MMR or were experts in MMR who had real-world MMR experiences. Fourth, we used the term “ethical conduct of research” as opposed to the more typical term for research ethics training, “responsible conduct of research” in our survey. Although this was an intentional term choice to make salient our focus on ethical issues within MMR research and while no respondent commented on the term, survey respondents may have been unsure about our specific meaning. We also acknowledge that our selection of eight ethical issues was intentionally focused on discrete activities (e.g., data management, recruitment) but these issues share content with the seven requirements for determining ethical clinical research proposed by Emanuel, Wendler, and Grady [20] (e.g., fair subject selection).

Our purposeful sample of mixed methods health services researchers with high reported levels of involvement with MMR offered the opportunity to capitalize on a wealth of practical experiences with MMR. Most reported training in research ethics, but only about one third rated that training as helpful in planning, conducting, or reporting MMR. While care must be taken making inferences from one question, that so few of such an experienced sample did not find training helpful is a challenge to make ethics training more salient for MMR. Research ethics training that incorporates MMR should pay special attention to issues that were rated as most challenging, including not only participant burden which is frequently mentioned in discussions of MMR [6], but also data management, communication and dissemination of MMR.

A notable finding was the limited reporting of mitigation strategies that we judged were specific to MMR and lack of comments on how any ethical issues might be intensified by use of mixed methods. Respondents appeared to mostly draw on strategies from either quantitative or qualitative traditions, but not in a “mixed” way. This may signal a need for dedicated empirical research and training related to ethical integrity-promoting strategies for MMR. MMR-specific strategies emphasized stakeholder engagement throughout the research process from participant recruitment through dissemination to different audiences. While stakeholder engagement is not exclusive to MMR, arguably one of the primary reasons for using mixed methods is to promote a deeper, richer understanding of experiences from multiple perspectives. With a key characteristic of integration of qualitative and quantitative research, MMR involves greater complexity than the use of either alone. The collection and integration of multiple data sources require unique ethical considerations when planning and may also increase the likelihood of ethical issues arising. Together, this suggests that ethics MMR training might be augmented by attention to how to meaningfully include multiple perspectives and stakeholder contributions to promote ethical conduct and dissemination of MMR.

Another unexpected finding was that respondents rated many ethical issues as not challenging to address despite occurring frequently. For example, more than 50% of respondents reported that issues related to protecting participant confidentiality frequently occurred in MMR but fewer than 25% of respondents reported that confidentiality issues were challenging to address, perhaps because strategies were rated as 75% effective (similar patterns were seen for informed consent and participant safety). We offer several possible explanations to understand these unexpected findings. It may be that ethical issues in MMR are indeed no more challenging than in single method research, or that there is limited awareness of how challenging health services MMR can be. On the other hand, ethical issues that were rated as most challenging with strategies rated as less effective (namely, communication and dissemination) may signal areas most in need of empirical ethics research or training support. In particular, communication within multidisciplinary teams and dissemination of MMR findings may be less familiar research practices, suggesting specific areas of dedicated attention for MMR training. Investigators may need to pay special attention to the most challenging issues when conducting MMR or training others. Another possibility is that this sample of researchers had experience conducting health services research before or in combination with MMR, providing them with greater practical experience navigating research ethics. Indeed, the sample was comprised of faculty (or equivalent research positions), more than half who were at the Associate or full Professor ranks.

## Conclusions

In drawing on the experiences of highly engaged and experienced investigators in health services MMR, our investigation identified what ethical issues are reported as most frequently encountered, perceived as challenging to mitigate, and effective strategies to address ethical issues. While we recognize our effort is only a start for empirical ethics research in MMR, we call upon others to build upon our emerging understandings and to generate empirical research to further our work. Our findings provide an essential beginning place for advancing implications salient to investigators, IRBs, and for mentoring and training in MMR. MMR is sometimes characterized as a “third” paradigm [22] beyond use of parallel quantitative and qualitative methods. As planning a research design demands investigators think in an integrated way, it may be useful to think of MMR ethics in an integrated way. Ensuring MMR expertise on review boards, acting in a collaborative, feedback-based fashion [23], would facilitate guidance from IRBs on navigating ethical issues in MMR health services research. Given the increasing number of MMR projects in health services and implementation science [19], applications to ethics review boards provide an opportunity to gather empirical evidence on what strategies to promote ethical MMR are most feasible and effective. Most responsible conduct of research programs required for conducting health research (e.g., [24]) focus on quantitative or clinical research methods with limited or no mention of mixed methods designs. Our findings highlight a need for both specialized MMR training programs and other health services research training programs to attend to ethical integrity from a mixed methods health services research perspective. As one of the first empirical studies to examine experiences with and managing ethical issues in health services MMR, our findings highlight the limited attention to and need for greater attention to ethics in health services MMR research and training.

## Supplementary Information

**Additional file 1.** Survey on ethics in mixed methods research in health.

## Data Availability

The datasets used and/or analyzed during the current study are available from the corresponding author on reasonable request.
