# Supplementary material for: Advancing discussion of ethics in mixed methods health services research
Source: BMC Health Serv Res. 2021 Jun 15;21:577. doi: 10.1186/s12913-021-06583-1 (PMC8204431; doi:10.1186/s12913-021-06583-1)
Supplement: Supplementary file 1 — Additional file 1. Survey on ethics in mixed methods research in health. [file 12913_2021_6583_MOESM1_ESM.docx]

Appendix: Survey on Ethics in Mixed Methods Research in Health

**Instructions:**
Thank you for taking the time to complete this survey! You were selected to complete this survey because you are a Scholar, Faculty or Consultant of the Mixed Methods Research Training Program in Health Sciences. Little empirical work on ethics in human subjects research has focused on mixed methods. We would like to use the information that you share to improve our understanding of successful strategies to address ethical issues when conducting mixed methods research. We are interested in your opinions and experiences. There are no right or wrong answers. The survey should take no more than 15 minutes to complete. If you are interested in learning about the results of this project, please enter your e-mail address at the end of the survey. Thank you!

Section I. About You

Q1 Please answer the following questions about your background and experience with mixed methods research.

Q2 What is your current title?_______________________________________________________________

Q3 Please identify your primary discipline:____________________________________________________________

Q4 Please answer the following questions about your background and experience with mixed methods research.

|  | Yes (5) | No (6) |
| --- | --- | --- |
| I am primarily trained in qualitative research. (1) |  |  |
| I am primarily trained in quantitative research. (2) |  |  |
| I am primarily trained in mixed methods research. (3) |  |  |
| I wrote a mixed methods research application that received funding. (4) |  |  |
| I have presented mixed methods research at a national or international meeting. (5) |  |  |
| I have published a paper using mixed methods. (6) |  |  |
| I wrote a thesis or dissertation involving mixed methods. (7) |  |  |
| I mentor or advise others in mixed methods research. (8) |  |  |

Q5 Other mixed methods research experience; please describe:

________________________________________________________________

Q6 I have completed training in ethical conduct of research.

- Yes (1)
- No (2)

Display This Question:

If I have completed training in ethical conduct of research. = Yes

Q7 Was this training mandatory?

- Yes (1)
- No (2)

Display This Question:

If I have completed training in ethical conduct of research. = Yes

Q8 To what extent do you agree with the following statements about the training that you completed in ethical conduct of research.

|  | Strongly disagree (1) | Disagree (2) | Agree (3) | Strongly agree (4) | Not applicable (5) |
| --- | --- | --- | --- | --- | --- |
| This training helped me think about **planning** mixed methods research. (1) |  |  |  |  |  |
| This training helped me think about **conducting** mixed methods research. (2) |  |  |  |  |  |
| This training helped me think about **reporting** the findings of mixed methods research. (3) |  |  |  |  |  |

Section II. Ethical Issues in Your Mixed Methods Research

Q9 We would like you to respond to the following based on **your experiences** planning and conducting mixed methods research.

|  | How **often** have you encountered the following ethical issues in planning and conducting your mixed methods research? | | | | | How **challenging** was it to address the following ethical issues in planning and conducting your mixed methods research? | | | |  |  |
| --- | --- | --- | --- | --- | --- | --- | --- | --- | --- | --- | --- |
|  | Never (1) | Rarely (2) | Often (3) | Always (4) | Not at all challenging (1) | | Somewhat challenging (2) | Challenging (3) | Very challenging (4) | | Not applicable (5) |
| Ensuring voluntary and informed **consent**. (1) |  |  |  |  |  | |  |  |  | |  |
| Ensuring equitable **recruitment** of participants including sample size considerations, saturation, power, and sampling. (2) |  |  |  |  |  | |  |  |  | |  |
| Ensuring participant **confidentiality** including linking data across data sources. (3) |  |  |  |  |  | |  |  |  | |  |
| Considering participant **burden** including time to complete research activities. (4) |  |  |  |  |  | |  |  |  | |  |
| Ensuring the **safety** and wellbeing of participants. (5) |  |  |  |  |  | |  |  |  | |  |
| **Data management** including the conduct of appropriate data analysis. (6) |  |  |  |  |  | |  |  |  | |  |
| Effective **communication** between and among the research team including relationships or partnerships among team members, including researchers and community partners who may have different codes of ethics. (7) |  |  |  |  |  | |  |  |  | |  |
| Appropriate and equitable **dissemination** including communicating results to diverse audiences, stakeholder involvement in data analysis and interpretation and providing respondents with results of study findings. (8) |  |  |  |  |  | |  |  |  | |  |

Skip To: End of Block If Now we would like for you to respond to the following questions based on your experiences plannin... : How often have you encountered the following ethical issues in planning and conducting your mixed... [ Never] (Count) = 8

Q10 We would like you to respond to the following based on **your experiences** planning and conducting mixed methods research.

|  | What strategies have you used to address the following ethical issues in your mixed methods research? If you have used multiple strategies, please describe the most effective strategy. | To what extent was this strategy that you described **effective** to address the following ethical issues in your mixed methods research? Please select the response that best matches your experience. | | | |  |  |
| --- | --- | --- | --- | --- | --- | --- | --- |
|  | Open text response (1) | N/A (1) | Not at all effective (2) | Somewhat effective (3) | Effective (4) | | Very Effective (5) |
| Ensuring voluntary and informed **consent**. (1) |  |  |  |  |  | |  |
| Ensuring equitable **recruitment** of participants including sample size considerations, saturation, power, and sampling. (2) |  |  |  |  |  | |  |
| Ensuring participant **confidentiality** including linking data across data sources. (3) |  |  |  |  |  | |  |
| Considering participant **burden** including time to complete research activities. (4) |  |  |  |  |  | |  |
| Ensuring the **safety** and wellbeing of participants. (5) |  |  |  |  |  | |  |
| **Data management** including the conduct of appropriate data analysis. (6) |  |  |  |  |  | |  |
| Effective **communication** between and among the research team including relationships or partnerships among team members, including researchers and community partners who may have different codes of ethics. (7) |  |  |  |  |  | |  |
| Appropriate and equitable **dissemination** including communicating results to diverse audiences, stakeholder involvement in data analysis and interpretation and providing respondents with results of study findings. (8) |  |  |  |  |  | |  |

Section III. Closing

Q11 Thank you for your time!

 If you provide us permission to contact you at a later date to expand upon your survey responses in an interview, please enter your e-mail address below. We anticipate that we will reach out to those interested in about 4-6 months. The interview will be conducted by phone and take approximately 30 minutes.

________________________________________________________________

Q12 If you would be interested in receiving updates about this project, please enter your e-mail address here:

________________________________________________________________

Q13 We would like to gather the perspectives of other researchers engaged in mixed methods research. We invite you to share this survey link with your colleagues: [LINK]

Thank you for your time in completing this survey! Have a great day!
